# Carboxypeptidase Z (CPZ) Links Thyroid Hormone and Wnt Signaling Pathways in Growth Plate Chondrocytes

**DOI:** 10.1359/jbmr.081014

**Published:** 2008-10-13

**Authors:** Lai Wang, Yvonne Y Shao, R Tracy Ballock

**Affiliations:** Orthopaedic Research Center, Departments of Orthopaedic Surgery and Biomedical Engineering, The Lerner Research Institute, The Cleveland Clinic Foundation Cleveland, Ohio, USA

**Keywords:** thyroid hormone, Wnt-4, carboxypeptidase Z, growth plate chondrocytes

## Abstract

Carboxypeptidase Z (CPZ) removes carboxyl-terminal basic amino acid residues, particularly arginine residues, from proteins. CPZ contains a cysteine-rich domain (CRD) similar to the CRD found in the frizzled family of Wnt receptors. We have previously shown that thyroid hormone regulates terminal differentiation of growth plate chondrocytes through activation of Wnt-4 expression and Wnt/β-catenin signaling. The Wnt-4 protein contains a C-terminal arginine residue and binds to CPZ through the CRD. The objective of this study was to determine whether CPZ modulates Wnt/β-catenin signaling and terminal differentiation of growth plate chondrocytes. Our results show that CPZ and Wnt-4 mRNA are co-expressed throughout growth plate cartilage. In primary pellet cultures of rat growth plate chondrocytes, thyroid hormone increases both Wnt-4 and CPZ expression, as well as CPZ enzymatic activity. Knockdown of either Wnt-4 or CPZ mRNA levels using an RNA interference technique or blocking CPZ enzymatic activity with the carboxypeptidase inhibitor GEMSA reduces the thyroid hormone effect on both alkaline phosphatase activity and Col10a1 mRNA expression. Adenoviral overexpression of CPZ activates Wnt/β-catenin signaling and promotes the terminal differentiation of growth plate cells. Overexpression of CPZ in growth plate chondrocytes also removes the C-terminal arginine residue from a synthetic peptide consisting of the carboxyl-terminal 16 amino acids of the Wnt-4 protein. Removal of the C-terminal arginine residue of Wnt-4 by site-directed mutagenesis enhances the positive effect of Wnt-4 on terminal differentiation. These data indicate that thyroid hormone may regulate terminal differentiation of growth plate chondrocytes in part by modulating Wnt signaling pathways through the induction of CPZ and subsequent CPZ-enhanced activation of Wnt-4.

## INTRODUCTION

Carboxypeptidases are zinc-containing exopeptidases that catalyze the removal of C-terminal amino acids from proteins and peptides. The members of the N/E subfamily of carboxypeptidases are selective enzymes relying on their substrate specificity and are thought to play a role in the processing of intercellular peptide messengers.(1) A well-studied N/E enzyme is carboxypeptidase N (CPN). CPN inactivates bradykinin and kallidin II by removal of their C-terminal arginine residues.(2) CPN also removes the C-terminal arginine from anaphylatoxin C3a, C4a, and C5a, which reduces their biological activities(3) and is responsible for the significant reduction in biological activity of chemokine SDF-1α that occurs through removal of its C-terminal lysine.(4)

Carboxypeptidase Z (CPZ) belongs to the N/E subfamily of carboxypeptidases. CPZ functions at neutral pH to remove basic amino acids, particularly arginine residues, from the carboxyl terminus of proteins.(1,5) CPZ contains a cysteine-rich domain (CRD) with 20–30% homology to the CRD present in the frizzled family of Wnt receptors, suggesting that CPZ may function as a modulator of Wnt signaling. A genomic analysis showed upregulation of CPZ expression along with activation of Wnt/β-catenin signaling during the alachlor-induced oncogenesis that occurs in rat olfactory mucosa.(6) The expression of CPZ also overlaps with the expression pattern of several Wnt genes and persists in cartilage condensations throughout mouse gestation.(7) Moeller et al.(8) showed expression of CPZ in the somites and sclerotome of the developing chicken embryo. Overexpression of CPZ in the chick somite induced the expression of the Wnt-responsive gene, *Pax3*, and resulted in dysmorphogesis of the scapula and ribs.

Wnt signaling has recently been recognized as an important signal transduction pathway in regulating chondrocyte proliferation and differentiation during limb development. The role of thyroid hormone in the regulation of skeletal development, particularly in the transition to the terminally differentiated growth plate chondrocyte, occurs in part through the modulation of canonical Wnt signaling.(9) Thyroid hormone may therefore exert its potent effect on maturation of the growth plate through activation of Wnt-4 expression and Wnt/β-catenin signaling.

Wnt proteins are locally released from cells and diffuse into the extracellular space. CPZ protein is also secreted and located in the extracellular matrix, suggesting that CPZ could directly interact with Wnt proteins. Given that Wnt-4 contains a C-terminal arginine, that CPZ binds to Wnt-4 through its CRD, and that CPZ enhances the Wnt-4–dependent induction of the homeobox gene *Cdx1*,(8) it is reasonable to postulate that CPZ may enhance Wnt-4 activity through enzymatic removal of its terminal arginine. The objective of this study therefore was to determine whether CPZ modulates Wnt/β-catenin signaling and terminal differentiation of growth plate chondrocytes.

## MATERIALS AND METHODS

### Cell culture

Chondrocytes were isolated from the distal femoral growth plate of 2-day-old neonatal Sprague-Dawley rats by sequential digestion in trypsin/EDTA (Invitrogen, Carlsbad, CA, USA) for 1 h at 37°C, followed by 0.3% collagenase type I (Worthington, Lakewood, NJ, USA) for 4 h at 37°C.(10) Cells were resuspended in DMEM/F12 medium (Invitrogen) supplemented with a defined media supplement (ITS+1; Sigma, St Louis, MO, USA) and plated in monolayer at a density of 2 × 10^5^ cells/cm^2^ or in 3D pellet culture at 2 × 10^5^ cells per 15-ml conical tube.(9) Triiodothyronine (T3; Sigma) was added to the medium at a concentration of 100 ng/ml. 2-guanidinoethylmercaptosuccinic acid (GEMSA; Calbiochem, La Jolla, CA, USA) was used as an inhibitor of carboxypeptidase activity.

An adenovirus was constructed (Vectorlab, Philadelphia, PA, USA) using a plasmid consisting of a long form of human CPZ cDNA containing the CRD, the carboxypeptidase domain, and a His-6 tag(11) (kindly supplied by Dr LD Fricker, Albert Einstein College of Medicine, Bronx, NY, USA). A similar adenovirus containing the CMV promoter was used as a negative control. Growth plate chondrocytes were plated in 60-mm dishes and infected with adenoviral vectors with a MOI (multiplicity of infection) of 100. The efficiency of the Ad-CPZ infection was confirmed by immunoblotting with anti-His-6 antibody. Twenty-four hours after infection, the cells in monolayer were trypsinized and maintained as pellet cultures in the presence or absence of T3.

### Laser capture microdissection

Frozen sections (10 μm thick) of 2-day-old rat distal femoral growth plate were cut on a cryostat, attached to foil mounted frame slides (Leica, Wetzkar, Germany), and stained with toluidine blue. Cell populations of resting zone (RZ), proliferating zone (PZ), prehypertrophic zone (preHZ), and hypertrophic zone (HZ) were collected using laser capture microdissection (LCM; Leica).

### Quantitative real-time PCR

Total RNA was isolated from cultured growth plate chondrocytes using the RNeasy Kit (Qiagen, Valencia, CA, USA) according to the manufacturer's instructions. Total RNA of microdissected growth plate cartilage was extracted using the RNeasy Micro Kit (Qiagen). Reverse transcription was performed using random primers and Superscript III DNA polymerase (Invitrogen). Real-time PCR reactions were conducted in an ABI Prism 7700 Sequence Detection System using SYBR Green PCR core reagents (Applied Biosystems, Foster City, CA, USA). The forward and reverse primers for the amplifications were as follows: CPZ, 5′-CCTTTGACGCCATCGACAT-3′ and 5′-GAGGGTCATAGCAGCCTTCCT-3′; Wnt-4, 5′-AACCGGCGCTGGAACTG-3′ and 5′-GGTCCCTTGTGTCACCACCTT-3′; Col10a1, 5′-GATCATGGAGCTCACGGAAAA-3′ and 5′-CCGTTCGATTCCGCATTG-3′; 18s, 5′-AGTCCCTGCCCTTTGTACACA-3′ and 5′-GATCCGAGGGCCTCACTAAAC-3′.

### Immunoblotting

Whole cell extracts were prepared from cultured chondrocytes using modified RIPA buffer (50 mM Tris-HCl, pH 7.4, 1% NP-40, 0.25% sodium deoxycholate, 150 mM NaCl, 1 mM EDTA, 1 mM PMSF, 1 μg/ml of each aprotinin, leupeptin, and pepstatin, 1 mM Na_3_VO_4_, 1 mM NaF). An equal amount of protein was separated by 10% SDS-PAGE and transferred onto nitrocellulose membranes. CPZ protein was detected using rabbit polyclonal antisera raised against the C-terminal 73 amino acids of rat CPZ (a gift from Dr Lloyd Fricker).(5) The cellular accumulation of β-catenin was detected using an antibody against β-catenin (Santa Cruz Biotechnology, Santa Cruz, CA, USA). Anti-β-actin (Sigma) was used as an internal control. The blots were incubated with a horseradish peroxidase (HRP)-conjugated secondary antibody (Santa Cruz). Immunoreactive proteins were visualized by Western blotting chemiluminescence luminol reagent (Santa Cruz).

For co-immunoprecipitation studies, cell lysates from growth plate chondrocytes transfected with a Wnt-4 expression plasmid and infected with Ad-CPZ were incubated with CPZ antisera for 2 h at 4°C, followed by an addition of protein A/G-agarose (Santa Cruz) for overnight incubation on a rotating device. The precipitated proteins were separated by SDS-PAGE and blotted onto nitrocellulose membranes. Immunodetection was carried out using an antibody against Wnt-4 (Zymed, South San Francisco, CA, USA) and an HRP-conjugated secondary antibody.

### CPZ enzymatic activity assay

Homogenates of growth plate chondrocyte pellets were assayed for carboxypeptidase activity using a dansyl-tagged peptide containing a C-terminal arginine (dansyl-Phe-Ala-Arg) as a substrate.(12) Cleavage of the C-terminal arginine results in a solubility change (i.e., the water-soluble dansyl-Phe-Ala-Arg is converted into the chloroform-soluble dansyl-Phe-Ala). Homogenates of growth plate cell pellets were incubated with 0.2 mM of dansyl-Phe-Ala-Arg in 100 mM Tris-Cl buffer (pH 7.4) in a final volume of 250 μl. After 2 h at 37°C, the reaction was terminated with 100 μl of 0.5 M HCl and 1 ml of chloroform was added. After mixing and centrifugation, fluorescence was measured in the chloroform phase and normalized to the total protein content in the homogenate.(13)

### Alkaline phosphatase activity assay

Alkaline phosphatase activity was measured in growth plate chondrocytes in pellet cultures. Pellets were homogenized, and alkaline phosphatase activity was determined as previously described using *p*-nitrophenol phosphate (Sigma) as a substrate.(10) One unit of alkaline phosphatase was defined as the enzyme activity that liberated 1 μmol *p*-nitrophenol per 30 min at 37°C/mg of protein.

### Transient transfections

TCF/LEF transcriptional activity was evaluated using the TCF Reporter Plasmid Kit from Upstate.(9) Chondrocytes were plated in monolayer culture and transfected using Fugene 6 (Roche, Indianapolis, IN, USA) and a TOPFlash reporter construct along with an internal control (phRG-TK; Promega, Madison, WI, USA). Twenty-four hours later, the transfection mixture was replaced with original medium with or without Ad-CPZ or Wnt-4–conditioned medium. After 48 h, cells were harvested and assayed for luciferase activity using the Dual-luciferase Reporter Assay System (Promega). The results were represented as the firefly luciferase relative light units (RLU) normalized to the *Renilla* luciferase RLU of the same sample.

RNA knockdown experiments were performed using RNA interference technique. The siRNA for Wnt-4 (NM_053402) and CPZ (NM_031766) were synthesized by Dharmacon (Lafayette, CO, USA) (siGENOME SMARTpool siRNA reagent, M-097135-00 and L-093743-01, respectively). siRNA against Wnt-4 or CPZ was transfected with Oligofectamine (Invitrogen) into growth plate chondrocytes cultured in monolayer according to the method of Elbashir et al.(14) siCONTROL from Dharmacon was used as a negative control. Quantitative RT-PCR was performed 36 h after transfection to confirm the knockdown of Wnt-4 and CPZ mRNA expression.

### MALDI-TOF spectroscopy analysis

Analysis of the Wnt-4 C-terminal peptide was performed on a Micromass TofSpec 2E matrix-assisted laser desorption/ionization time-of-flight (MALDI-TOF) mass spectrometer. Freshly isolated growth plate chondrocytes were plated in 60-mm culture dishes in DMEM/F12 supplemented with ITS+. The cells were infected with Ad-CPZ or control Ad-Gal at an MOI of 100 for 48 h. Two hundred microliters of the cell homogenates was incubated with 25 μl of a synthetic peptide consisting of the 16 C-terminal amino acids of the Wnt-4 protein (VKCRQCQRLVEMHTCR) at a concentration of 0.5 mM in 100 mM Tris-Cl buffer (pH 7.4) in a final volume of 250 μl. After 2-h incubation at 37°C, the reaction was terminated with 100 μl of 0.5 M HCl. The samples were diluted 1:2 in acetonitrile, and a 1.5-μl aliquot of the diluted sample was added to 18.5 μl of matrix (α-cyano-4-hydroxycinnamic acid, 10 mg/ml in acetonitrile/ethanol/water 4/4/2). The MALDI spectra were acquired in the reflectron mode, using a constant laser power. Each spectrum was generated by combining the data from ∼200 laser shots.

### Site-directed mutagenesis

Truncated Wnt-4 lacking the C-terminal arginine was created using the QuickChange II Site-directed Mutagenesis Kit (Stratagene, La Jolla, CA, USA) according to the manufacturer's instructions. HA-tagged mouse Wnt4 cDNA in pUSEamp was purchased from Upstate Biotechnology (Charlottesville, VA, USA). The primers used to amplify wildtype Wnt-4 without the HA-tag were 5′-GCACACGTGCCGGTAATTAATTAAGATCCGG-3′ and 5′-CCGGATCTTAATTAATTACCGGCACGTGTGC-3′. The primers used to create truncated Wnt-4 were 5′-GCACACGTGCTAATTAATTAAGATCCGGCTC-3′ and 5′-GAGCCGGATCTTAATTAATTAGCACGTGTGC-3′ (underlined letters represent the mutation of the complementary primers). To create the truncated Wnt-4 cDNA, the nucleotide coding sequence for the C-terminal arginine in the mouse Wnt-4 cDNA (CGG) was deleted and replaced with a premature stop codon (TAA).

**FIG. 6:**
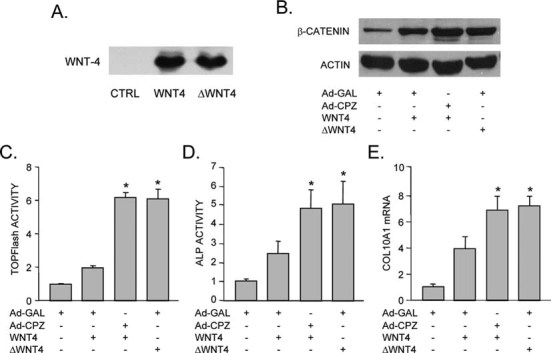
Both overexpression of CPZ and removal of the Wnt-4 C-terminal arginine promote Wnt4-activated Wnt/β-catenin signaling and terminal differentiation of growth plate chondrocytes. Growth plate cells were treated with Wnt-4–conditioned medium in the presence or absence of infection with Ad-CPZ (MOI of 100) or treated with C-terminal arginine-truncated Wnt-4 (ΔWnt-4)–conditioned medium. (A) Immunoblotting of Wnt-4 protein levels in conditioned medium from HEK293 cells transfected with wildtype or truncated Wnt-4 or empty plasmid. (B) Immunoblotting of the cell lysates after overexpression of Wnt-4, Wnt-4 and CPZ, or ΔWnt-4 for 5 days. (C) TCF/LEF transcriptional activity in cells treated with Wnt-4, Wnt-4 and CPZ, or ΔWnt-4 for 48 h. (D) Alkaline phosphatase activity in cells treated with Wnt-4, Wnt-4 and CPZ, or ΔWnt-4 for 5 days. (E) The cells treated with Wnt-4, Wnt-4 and CPZ, or ΔWnt-4 for 5 days were analyzed for Col10a1 mRNA expression, which was normalized to the expression of Wnt-4 (including both Wnt-4 and ΔWnt-4) in the same cells. The data were expressed as the fold over the control cells without CPZ, Wnt-4, or ΔWnt-4 treatment (*p < 0.05 vs. cells treated with full-length Wnt-4–conditioned medium alone).

To generate Wnt-4–conditioned medium, wildtype and truncated Wnt-4 plasmids were transiently transfected into HEK293 cells and cultured in DMEM containing 10% FBS. Control medium was obtained from HEK293 cells transfected with empty pcDNA3 plasmid under the same conditions. Medium was collected 48 h after transfection. Protein levels of Wnt-4 in the conditioned medium were examined by immunoblotting using antibody recognizing both wildtype and truncated Wnt-4 (Zymed) (Fig. 6A). Wnt-4–conditioned medium or control medium was added to the pellet cultures in a 1:5 ratio by volume of DMEM/F12 containing ITS+.

### Statistical analysis

The data are represented as mean ± SD of triplicate samples from at least three independent experiments. Values were assessed by one-way ANOVA with the Bonferroni posthoc test and Student's *t*-test at a significance level of *p* < 0.05.

## RESULTS

### Wnt-4 and CPZ expression co-localize in the growth plate

**FIG. 1:**
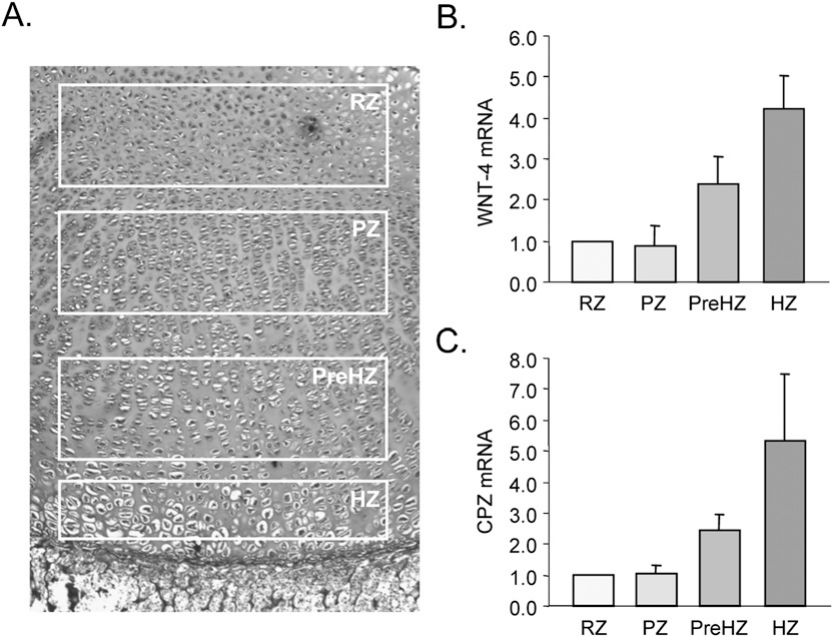
Wnt-4 and CPZ are co-expressed in growth plate chondrocytes. (A) Photomicrograph of a toluidine blue-stained histologic section of the 2-day-old rat distal femoral growth plate, showing the regions of resting zone (RZ), proliferating zone (PZ), prehypertrophic zone (PreHZ), and hypertrophic zone (HZ) captured by laser capture microdissection. (B and C) Quantitative real-time RT-PCR analysis of expression of Wnt-4 mRNA (B) and CPZ mRNA (C) in laser capture microdissected cells from RZ, PZ, preHZ, and HZ of growth plate cartilage. The expression levels were normalized to the expression in RZ cells.

Combining the LCM technique with quantitative real-time PCR analysis allowed the mRNA expression in the different cell populations of the growth plate to be analyzed (Fig. 1A). Increased expression of both Wnt-4 and CPZ mRNA was observed in the prehypertrophic and hypertrophic zones of the distal femoral growth plate compared with the resting and proliferating zone (Figs. 1B and 1C).

### T3 upregulates CPZ expression and increases CPZ enzymatic activity

**FIG. 2:**
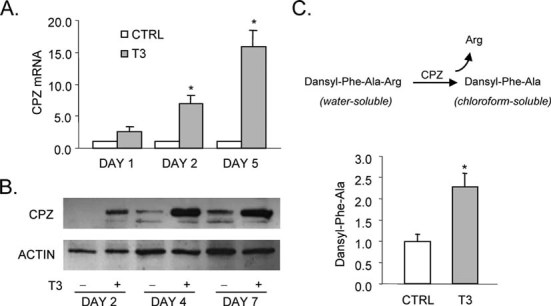
Thyroid hormone treatment increases CPZ expression and CPZ carboxypeptidase activity in rat growth plate chondrocytes. (A) Quantitative real-time PCR analysis of CPZ mRNA expression in growth plate chondrocyte pellet cultures treated with T3 (100 ng/ml) for 1–5 days. The expression of CPZ in T3-treated cells was normalized to the expression in T3-untreated cells (*p < 0.05 vs. the T3-untreated cells). (B) Immunoblotting of CPZ protein from whole cell lysates of growth plate chondrocytes treated with or without T3 (100 ng/ml) for 2–7 days. Actin was used as an internal control. (C) CPZ carboxypeptidase activity in growth plate chondrocytes treated with T3 (100 ng/ml) for 5 days. Cleavage of Dansyl-Phe-Ala from Dansyl-Phe-Ala-Arg was measured in the chloroform phase and normalized to the total protein content in the homogenate (*p < 0.05 vs. the T3-untreated cells).

CPZ mRNA expression was examined by quantitative real-time PCR analysis of total RNA extracted from resting zone growth plate chondrocytes treated with thyroid hormone for 1–5 days. CPZ expression was increased as early as day 2 in pellet cultures of growth plate cells treated with T3, with a 16-fold increase in CPZ expression observed after 5 days of T3 treatment (Fig. 2A).

To confirm that the increase in CPZ gene expression indeed resulted in a corresponding increase in CPZ protein, anti-sera raised to the carboxyl terminus of CPZ were used for immunoblotting of cell lysates from the growth plate cells treated with T3 for 2–7 days. Figure 2B shows a corresponding increase in immunoreactive CPZ with the correct molecular weight (71 kDa) in response to T3 treatment.

Carboxypeptidase enzymatic assays using the substrate dansyl-Phe-Ala-Arg at pH 7.4 showed increased CPZ activity in homogenates from growth plate cells treated with T3 for 5 days compared with homogenates from controls without T3 treatment (Fig. 2C).

### Knockdown of either Wnt-4 or CPZ expression or inhibition of CPZ enzymatic activity interferes with T3-induced terminal differentiation

**FIG. 3:**
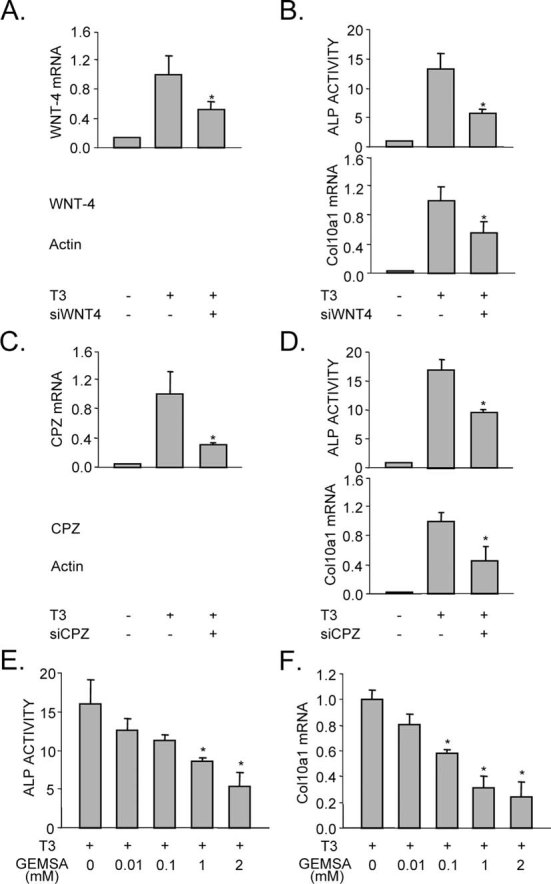
T3-induced increases in terminal differentiation of growth plate chondrocytes are suppressed by either knockdown of Wnt-4 or CPZ mRNA expression or by inhibition of CPZ enzymatic activity. (A) Wnt-4 mRNA expression and Wnt-4 protein levels in growth plate chondrocytes subjected to Wnt-4 mRNA knockdown and treatment with T3 for 5 days (*p < 0.05 vs. the cells treated with control siRNA and T3). (B) Alkaline phosphatase activity and expression of Col10a1 mRNA in growth plate chondrocytes subjected to Wnt-4 knockdown and T3 treatment for 5 days (*p < 0.05 vs. the cells treated with control siRNA and T3). (C) CPZ mRNA expression and CPZ protein levels in growth plate chondrocytes subjected to CPZ mRNA knockdown and treatment with T3 for 5 days (*p < 0.05 vs. cells treated with control siRNA and T3). (D) Alkaline phosphatase activity and expression of Col10a1 mRNA in growth plate chondrocytes subjected to CPZ knockdown and T3 treatment for 5 days (*p < 0.05 vs. cells treated with control siRNA and T3). (E and F) Alkaline phosphatase activity (E) and Col10a1 mRNA (F) of growth plate chondrocytes treated for 5 days with T3 and the carboxypeptidase inhibitor GEMSA (*p < 0.05 vs. cells treated with T3 alone).

Wnt-4 or CPZ RNA knockdown experiments were performed using an siRNA approach. Quantitative RT-PCR showed that transfection with anti-Wnt4 or anti-CPZ siRNA resulted in 50–80% suppression of gene expression after 36 h of treatment at a final concentration of 100 nM (data not shown). Figure 3A shows that Wnt-4 mRNA expression after 5 days of T3 treatment was reduced by 50% by the anti-Wnt4 siRNA. T3-induced CPZ mRNA expression was reduced by 70% by knockdown with anti-CPZ siRNA (Fig. 3C). T3-induced increases of Wnt-4 or CPZ protein levels were also attenuated by anti-Wnt4 or anti-CPZ siRNA, respectively (Figs. 3A and 3C).

Analysis of markers of chondrocyte terminal differentiation showed that T3-induced increases in alkaline phosphatase enzymatic activity and Col10a1 mRNA expression (Figs. 3B and 3D) were decreased by ∼50% in the Wnt-4 or CPZ knockdown cells compared with controls.

GEMSA has been previously shown to be a potent inhibitor of CPZ enzymatic activity in an in vitro assay using purified CPZ protein.(13) Inhibition of CPZ activity by GEMSA significantly reduced the T3-induced increases in alkaline phosphatase activity and Col10a1 mRNA expression by growth plate chondrocytes in a dose-dependent manner at concentrations ranging from 0.01 to 2 mM (Figs. 3E and 3F).

### CPZ binds to Wnt-4 and removes its C-terminal arginine

**FIG. 4:**
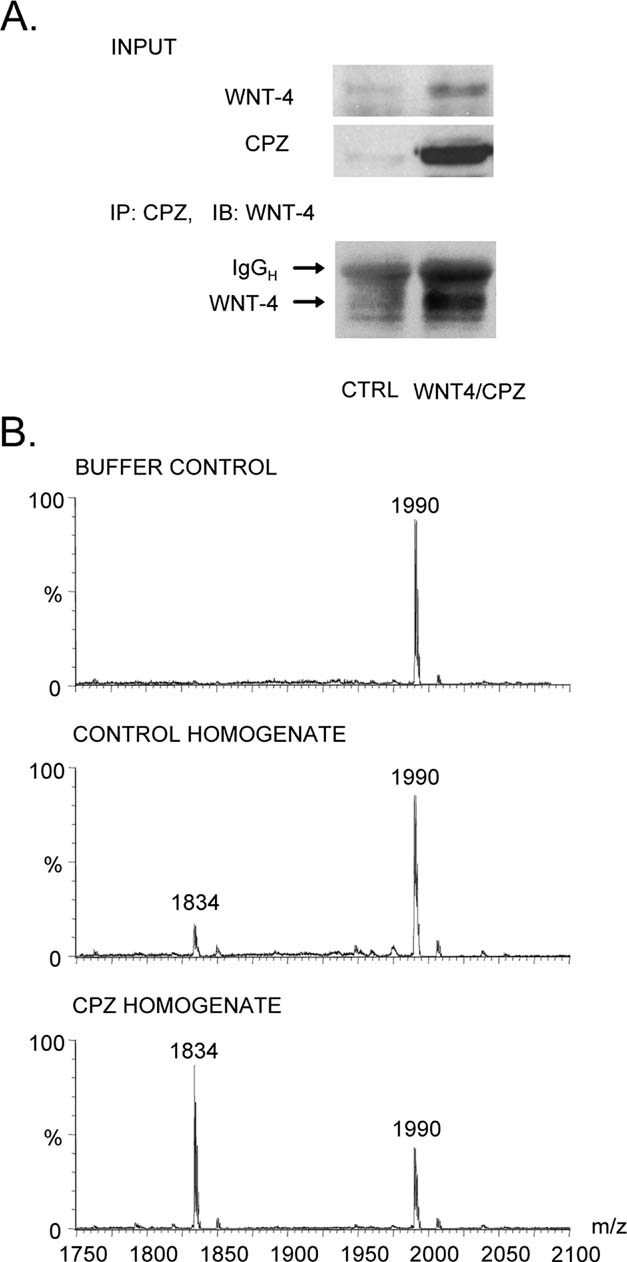
CPZ binds to Wnt-4 and removes its C-terminal arginine in growth plate chondrocytes. (A) Co-immunoprecipitation and immunoblotting. Cell lysates from growth plate chondrocytes transfected with a Wnt-4 expression plasmid and infected with Ad-CPZ for 2 days were immunoprecipitated (IP) with CPZ anti-sera and immunoblotted using an anti-Wnt-4 antibody. Wnt-4 and CPZ were also detected in the input lysates (5% of the input for immunoprecipitation). (B) MALDI-TOF mass spectrometric analysis. A synthetic peptide consisting of the C-terminal 16 amino acids of Wnt-4 was incubated at 37°C for 2 h with control buffer or homogenates of growth plate chondrocytes infected with or without Ad-CPZ. The peak at 1834 indicates the position of the 15-aa peptide lacking the C-terminal arginine, whereas the peak at 1990 indicates the position of the 16-aa peptide containing the C-terminal arginine.

For detection of binding of CPZ to Wnt-4 in growth plate chondrocytes, cells were transfected with a Wnt-4 expression plasmid and treated with Ad-CPZ for 2 days. Co-immunoprecipitation and immunoblotting of the lysates confirmed the physical association of Wnt-4 with CPZ (Fig. 4A).

The interactions between CPZ and Wnt-4 were further analyzed using MALDI-TOF spectroscopy. Homogenates of growth plate chondrocytes treated with Ad-CPZ were collected and incubated with a synthetic peptide consisting of the 16 C-terminal amino acid residues of Wnt-4 (16-aa, VKCRQCQRLVEMHTCR, with a calculated mass of 1990 Da). Analysis of the samples by MALDI showed a relative molecular mass of 1834 Da after incubation of 16-aa with CPZ-containing homogenates, corresponding to the predicted position of the 15 amino acid fragment (15-aa, VKCRQCQRLVEMHTC) that remains after cleavage of the C-terminal arginine by CPZ (Fig. 4B). The peak at 1834 observed in control homogenate was attributed to endogenous CPZ in chondrocytes.

### CPZ promotes Wnt signaling and terminal differentiation of growth plate chondrocytes

**FIG. 5:**
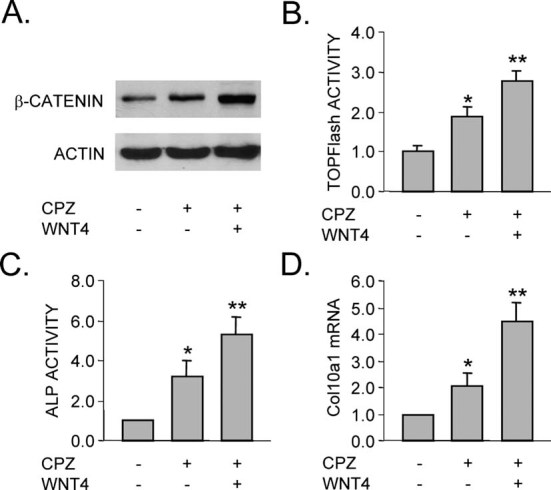
CPZ promotes and Wnt-4 enhances terminal differentiation of growth plate chondrocytes. (A) Immunoblotting of β-catenin from whole cell lysates of growth plate chondrocytes infected with Ad-CPZ (MOI of 100) for 4 days in the absence or presence of Wnt-4–conditioned medium. Actin was used as an internal control. (B) TCF/LEF transcriptional activity (TOPFlash) of growth plate chondrocytes infected with Ad-CPZ for 48 h in the absence or presence of Wnt-4–conditioned medium. (C and D) Alkaline phosphatase activity (C) and Col10a1 mRNA expression (D) in growth plate chondrocytes infected with Ad-CPZ for 4 days in the absence or presence of Wnt-4–conditioned medium. The data are expressed as the fold increase over the control Ad-Gal-infected cells (*p < 0.05 vs. control cells infected with Ad-Gal; **p < 0.05 vs. cells infected with Ad-CPZ).

Adenoviral overexpression of CPZ increased both the cellular accumulation of β-catenin (Fig. 5A) and TCF/LEF transcriptional activity (Fig. 5B) in growth plate chondrocytes. Both alkaline phosphatase activity and Col10a1 mRNA were also increased after infection with Ad-CPZ at an MOI of 100 (Figs. 5C and 5D). These effects of CPZ alone on chondrocyte Wnt signaling and terminal differentiation were significantly enhanced by co-addition of Wnt-4–conditioned medium (Figs. 5A–5D).

### CPZ enhances the Wnt-4 activity in growth plate chondrocytes

To study the possible role of CPZ and Wnt-4 interaction in the regulation of the Wnt signaling pathway, growth plate cells were treated with Wnt-4–conditioned medium alone or in combination with Ad-CPZ. The combined treatment of Wnt-4–conditioned medium and CPZ adenovirus increased the cellular accumulation of β-catenin and TCF/LEF transcriptional activity in growth plate cells compared with treatment with Wnt-4 alone (Figs. 6B and 6C) and also produced significantly greater levels of alkaline phosphatase activity and Col10a1 mRNA expression than Wnt-4 alone after 5 days of treatment (Figs. 6D and 6E). The increases in TCF/LEF transcriptional activity, alkaline phosphatase activity, and Col10a1 mRNA expression induced by the combination of CPZ and Wnt-4 were significantly greater than in control cells without CPZ and Wnt-4 treatment.

### Cleavage of the C-terminal arginine of Wnt-4 enhances Wnt-4 activity

To determine whether Wnt-4 is a potential target of CPZ activity in the growth plate, a truncated Wnt-4 (ΔWnt-4) expression vector lacking the C-terminal arginine was constructed by site-directed mutagenesis. This change in the cDNA sequence was confirmed by DNA sequencing of the plasmid.

Compared with full-length Wnt-4, incubation of growth plate cells with the truncated Wnt-4 construct induced an increased cellular accumulation of β-catenin (Fig. 6B) and TCF/LEF transcriptional activity (Fig. 6C) and was more potent in increasing alkaline phosphatase activity (Fig. 6D) and Col10a1 mRNA expression (Fig. 6E) than the wildtype Wnt-4. The effects of truncated Wnt-4 without C-terminal arginine were comparable to the effects of the combination of wildtype full-length Wnt4 and CPZ treatment from the same group of cell cultures at the same time point.

## DISCUSSION

Wnt-4 expression is upregulated by thyroid hormone in growth plate chondrocytes(9) and has been discovered to act as a positive regulator of terminal differentiation of growth plate chondrocytes during skeletal development. In a chick model, Wnt-4 blocks the initiation of chondrogenesis and accelerates terminal chondrocyte differentiation.(15–17) Conditional expression of Wnt-4 during chondrogenesis also leads to dwarfism in mice through inhibition of growth plate chondrocyte proliferation and promotion of hypertrophy.(18) In this study, we confirmed that Wnt-4 plays a key role in the thyroid hormone-mediated terminal differentiation of growth plate chondrocytes. This study also identifies CPZ as an important enzyme that is able to remove the C-terminal arginine from Wnt-4 in growth plate chondrocytes, and this removal not only increases Wnt-4 activity, but also further promotes terminal chondrocyte differentiation. These findings indicate that the potent and positive thyroid hormone effect on skeletal maturation may result in part from CPZ-mediated activation of Wnt signaling in the growth plate. It also suggests a model of thyroid hormone action in which thyroid hormone initially upregulates the expression of both Wnt-4 and CPZ. CPZ subsequently removes the C-terminal arginine from Wnt-4, thereby enhancing the activity of Wnt-4 in stimulating Wnt/β-catenin signaling and further promoting the terminal differentiation of growth plate chondrocytes.

Novikova et al.(7) examined the distribution of CPZ throughout mouse embryonic development and found that CPZ is expressed in mouse cartilage condensations at E17, and that, in general, CPZ expression in adult tissues is much less abundant than in embryonic organs. The expression pattern of CPZ overlaps with the expression pattern of several Wnt genes, which are also maximally expressed during mouse embryonic development but expressed in a more restricted fashion in adult tissue.

Wnt-4 is the only known Wnt ligand that contains a predicted C-terminal arginine residue in both rat and mouse species. In the chick, both Wnt-4 and Wnt-8c have predicted C-terminal arginine residues, and interestingly, also are the only two Wnts known to induce terminal differentiation of growth plate chondrocytes.(17) Wnt-8c, which is not expressed in mice, rats, or humans, actually has arginine residues in the last two carboxyl positions.(19) The single C-terminal arginine is conserved in human, mouse, rat, and chick Wnt-4 proteins, whereas none of the other Wnts (except Wnt-9b in the mouse) exhibit this feature. This suggests the possibility that the C-terminal arginine residue may be important in the transduction of the Wnt signal that enhances terminal differentiation of growth plate cells.

The presence of the CRD domain within the CPZ protein and the co-localization of CPZ expression with Wnt-4 expression in the growth plate suggest that CPZ functions as a Wnt-binding protein. During canonical Wnt signaling, Wnt proteins serve as ligands for the Frizzled family of transmembrane receptors and act through the β-catenin pathway to regulate gene expression. The cysteine-rich Wnt binding domain does not occur only in Frizzled proteins but is also found in secreted Frizzled-related proteins (sFRPs), which antagonize Wnt action by competing with the Frizzled transmembrane receptors for Wnt binding. Although CPZ also harbors a CRD domain, unlike the sFRPs which inhibit Wnt signaling, CPZ binds to Wnt-4 and enhances its activity by proteolytically removing the arginine at the C terminus. Therefore, we postulate that the catalytic domain in CPZ, as opposed to the CRD domain, plays the pivotal role in the modulation of Wnt function. However, binding to Wnt-4 through the CRD may be needed to allow the catalytic action of CPZ to occur.

We conclude from these studies that CPZ may act as a critical link between the thyroid hormone and Wnt signaling pathways in the growth plate. It is possible that the carboxypeptidase domain of CPZ cleaves substrates other than the Wnt-4 protein in promoting Wnt signaling and chondrocyte terminal differentiation. For example, in the chick model, Wnt-8c might be another target of CPZ. Nonetheless, these studies identify CPZ as an enzyme capable of regulating Wnt-4 activity in the terminal differentiation of rat growth plate chondrocytes. These findings not only have significant implications for our understanding of skeletal biology of the growth plate but also raise the possibility that CPZ-mediated Wnt activation might be a general mechanism of thyroid hormone action during development.
